# Ambient Ozone Concentrations and the Risk of Perforated and Nonperforated Appendicitis: A Multicity Case-Crossover Study

**DOI:** 10.1289/ehp.1206085

**Published:** 2013-07-11

**Authors:** Gilaad G. Kaplan, Divine Tanyingoh, Elijah Dixon, Markey Johnson, Amanda J. Wheeler, Robert P. Myers, Stefania Bertazzon, Vineet Saini, Karen Madsen, Subrata Ghosh, Paul J. Villeneuve

**Affiliations:** 1 Department of Medicine,; 2 Department of Community Health Sciences, Environmental Health Research Group, Institute of Public Health, and; 3 Department of Surgery, University of Calgary, Calgary, Alberta, Canada; 4 Air Health Science Division, Health Canada, Ottawa, Ontario, Canada; 5 Department of Geography, and; 6 Faculty of Veterinary Medicine, University of Calgary, Calgary, Alberta, Canada; 7 Department of Medicine, University of Alberta, Edmonton, Alberta, Canada; 8 Population Studies Division, Health Canada, Ottawa, Ontario, Canada; 9 Institute of Health: Science, Technology and Policy, Carleton University, Ottawa, Ontario, Canada

**Keywords:** air pollution, appendicitis, environmental health, risk factors

## Abstract

Background: Environmental determinants of appendicitis are poorly understood. Past work suggests that air pollution may increase the risk of appendicitis.

Objectives: We investigated whether ambient ground-level ozone (O_3_) concentrations were associated with appendicitis and whether these associations varied between perforated and nonperforated appendicitis.

Methods: We based this time-stratified case-crossover study on 35,811 patients hospitalized with appendicitis from 2004 to 2008 in 12 Canadian cities. Data from a national network of fixed-site monitors were used to calculate daily maximum O_3_ concentrations for each city. Conditional logistic regression was used to estimate city-specific odds ratios (ORs) relative to an interquartile range (IQR) increase in O_3_ adjusted for temperature and relative humidity. A random-effects meta-analysis was used to derive a pooled risk estimate. Stratified analyses were used to estimate associations separately for perforated and nonperforated appendicitis.

Results: Overall, a 16-ppb increase in the 7-day cumulative average daily maximum O_3_ concentration was associated with all appendicitis cases across the 12 cities (pooled OR = 1.07; 95% CI: 1.02, 1.13). The association was stronger among patients presenting with perforated appendicitis for the 7-day average (pooled OR = 1.22; 95% CI: 1.09, 1.36) when compared with the corresponding estimate for nonperforated appendicitis [7-day average (pooled OR = 1.02, 95% CI: 0.95, 1.09)]. Heterogeneity was not statistically significant across cities for either perforated or nonperforated appendicitis (*p* > 0.20).

Conclusions: Higher levels of ambient O_3_ exposure may increase the risk of perforated appendicitis.

## Introduction

The lifetime risk of appendicitis is approximately 1 in 15, and appendectomy for appendicitis is among the most frequently performed operations in developed nations (Hardin 1999). Perforated appendicitis has a worse prognosis than nonperforated because of its increased risks of sepsis and mortality (Hardin 1999). Perforated appendicitis may result from delayed treatment (Bickell et al. 2006), but others have suggested that it may be a distinct phenotype with diverging pathogenesis from nonperforated appendicitis (Andersson 1999; Livingston et al. 2007; Ruber et al. 2006, 2010). In the United States, appendicitis-related hospitalizations contribute to approximately US$3 billion in hospital charges annually (Davies et al. 2004). Thus, appendicitis is a relatively common disease that imparts a significant burden to patients and to the health care system (Davies et al. 2004).

Despite the health and economic impacts of this disease, the pathogenesis of appendicitis remains largely unknown. Consistent epidemiological features of appendicitis include an elevated risk associated with younger age, male sex, Caucasian race, and warmer seasons (Addiss et al. 1990; Luckmann and Davis 1991). Additionally, temporal–spatial clustering of appendicitis cases has been reported, suggesting that acute environmental exposures might contribute to the pathogenesis of appendicitis (Andersson et al. 1995).

A decrease in the incidence of appendicitis in developed countries during the latter part of the 20th century (Addiss et al. 1990; Ferris et al. 2010) coincided with the enactment of legislation that led to reductions in the concentrations of several outdoor air pollutants (Chen et al. 2007). This motivated a previous study of short-term changes in air pollution and appendicitis in Calgary, Alberta, which indicated that the 7-day average concentration of ambient ozone (O_3_) was positively associated with appendicitis (Kaplan et al. 2009). Further, several recent animal studies have shown that air pollution exposure may alter intestinal immunity, increase gut permeability, and influence intestinal microbial composition (Kaplan et al. 2012; Kish et al. 2013; Mutlu et al. 2011). Such effects might predispose certain individuals to develop appendicitis, or might influence the clinical presentation of appendicitis.

To our knowledge, there has been no attempt to replicate our previously reported association between O_3_ exposure and appendicitis (Kaplan et al. 2009), or to evaluate associations according to appendicitis presentation (i.e., perforated vs. nonperforated appendicitis). Therefore, we conducted a multicity population-based case-crossover study of appendicitis patients to estimate associations between short-term ambient O_3_ concentrations and appendicitis across multiple Canadian cities. In addition, we examined whether associations varied between perforated and nonperforated appendicitis cases.

## Methods

*Study population*. We used the Discharge Abstract Database, maintained by the Canadian Institute for Health Information (CIHI; Ottawa, Ontario, Canada), to identify patients hospitalized with appendicitis (Lalonde and Taylor 1997). This database includes all inpatient discharges from nine provinces and two territories (Quebec excluded) (Lalonde and Taylor 1997). Patients hospitalized with appendicitis between 1 January 2004 and 31 December 2008 were identified for the following 12 cities: Vancouver, Calgary, Edmonton, Saskatoon, Regina, Winnipeg, Windsor, London, Hamilton, Toronto, Ottawa, and Halifax. These cities were selected because of their large populations and the availability of appendicitis data and daily O_3_ monitoring data collected by Environment Canada (Gatineau, Quebec, Canada) for > 80% of the study days. Appendicitis patients living outside city boundaries were identified based on their postal code and excluded from the analysis.

Each incident case of appendicitis was identified by a diagnostic code for nonperforated appendicitis [*International Classification of Diseases, 10th Revision, Canada* (CIHI 2013) ICD-10-CA code K35.9] or perforated appendicitis (ICD-10-CA codes K35.0 and K35.1) and a concurrent procedural code for appendectomy (Canadian Classification of Health Interventions, 1.NV.89.DA and 1.NV.89.LA) (CIHI 2013). Individuals coded with unspecified or other appendicitis (e.g., chronic or recurrent appendicitis) were excluded. Sensitivity and positive predictive value of ICD-10-CA coding for appendicitis were 94% and 85%, respectively (Kareemi et al. 2012).

*Air pollution exposure assessment*. Environment Canada’s National Air Pollution Surveillance (NAPS) network monitors ambient O_3_ levels in > 150 stations in 55 cities across Canada. Automated fixed-site continuous monitoring stations collect hourly mean concentration data that are used to calculate daily maximum O_3_ concentrations. When there were multiple monitors in a given city, O_3_ concentrations were averaged into a daily value for the city (Sajani et al. 2010). In addition, daily mean concentrations of nitrogen dioxide (NO_2_) and particulate matter with an aerodynamic diameter of ≤ 2.5 μm (PM_2.5_) were determined using data from fixed monitoring sites. Data for daily mean temperature and relative humidity were also provided by Environment Canada.

*Study design*. Associations between ambient O_3_ concentrations and appendicitis were investigated using a time-stratified case-crossover study design (Schwartz 2004). This design is an adaptation of the case–control study in which cases serve as their own controls (Maclure 1991). For each case of appendicitis, air pollution exposure on the ‘‘index’’ day (i.e., the day of appendicitis admission) is compared to exposure on a series of referent days that occur on the same day of the week during the same month and year as the index day. Because all comparisons are within-individuals, confounding by individual-level risk factors is controlled by the design: These factors are not expected to vary within the 1-month time frame that includes the index and referent days (e.g., genetics, obesity) (Levy et al. 2001; Schwartz 2004). Selecting referent intervals close in time to the case event also controls for seasonal patterns in disease occurrence. Although there is variation in the number of referent days that occur after or before the case event, over all appendicitis cases, the numbers of referent days before and after case events are comparable and thus there is no bias resulting from time trends (Janes et al. 2005; Levy et al. 2001; Schwartz 2004).

*Statistical analysis*. Associations between ambient O_3_ concentrations and appendicitis were examined using the 1-hr daily maximum O_3_ concentrations on the same or previous day and also using the average of 1-hr daily maximum O_3_ concentrations over the 3, 5, or 7 days prior to the event day or referent days, not including the event or referent days. The 3-, 5-, and 7-day averages of O_3_ were previously shown to be associated with appendicitis (Kaplan et al. 2009) and were therefore identified *a priori* as the primary exposures of interest. Conditional logistic regression was used to estimate the odds of appendicitis in relation to an interquartile range (IQR) increase in the daily maximum O_3_ concentration adjusted for mean temperature and relative humidity on the event or referent day. The IQR (16 ppb) was based on daily 1-hr maximum O_3_ levels throughout the entire study period (1 January 2004–31 December 2008) (Table 1). City-specific odds ratios (ORs) and 95% confidence intervals (CIs) were pooled using a random effects meta-analysis, which is less prone to bias due to heterogeneity. Heterogeneity in OR estimates across the cities was evaluated using Cochran’s Q statistic and quantified using *I*^2^.

**Table 1:** City-specific characteristics of patients with appendicitis and daily O_3_ levels in 12 Canadian cities, 2004–2008.

City	Daily 1-hr maximum O_3_ (ppb) [median (25th–75th percentile)]	Daily O_3_ (ppb) (range)	No. of monitors per city	Population size^*a*^	No. of appendicitis cases^*b*^	Age (years) [median (IQR)]	Percent female	Percent perforated appendicitis
Vancouver, British Columbia	29.3 (22.3–35.6)	2.3–75.1	17	578,041	3,385	33 (22–48)	47	37
Edmonton, Alberta	35.6 (28.4–44.6)	5.9–74.0	9	730,372	3,155	29 (20–45)	46	35
Calgary, Alberta	34.7 (27.7–42.0)	6.5–69.7	8	988,193	5,299	29 (19–44)	45	31
Saskatoon, Saskatchewan	30.0 (24.0–38.0)	5.0–64.0	1	202,340	961	27 (19–43)	46	31
Regina, Saskatchewan	34.5 (28.5–41.0)	6.5–66.0	2	179,246	871	28 (18–44)	45	31
Winnipeg, Manitoba	30.5 (24.0–38.0)	6.0–79.5	2	633,451	2,482	28 (18–45)	45	31
Ottawa, Ontario	34.0 (27.0–42.0)	1.0–86.5	3	812,129	3,149	30 (19–45)	46	28
Toronto, Ontario	35.5 (27.3–46.2)	5.7–96.3	7	2,503,281	9,564	31 (20–45)	45	31
London, Ontario	37.0 (28.0–48.5)	3.0–93.0	1	352,395	1,679	28 (17–44)	46	24
Windsor, Ontario	39.3 (28.0–53.3)	1.5–117.7	3	216,473	868	30 (18–45)	43	29
Hamilton, Ontario	37.0 (29.0–49.0)	5.0–101.5	2	504,559	2,922	30 (17–46)	47	31
Halifax, Nova Scotia	27.0 (21.0–34.0)	3.0–93.0	2	372,679	1,476	30 (19–45)	44	23
Overall	33.3 (26.0–42.0)	1.0–117.7	57	8,073,159	35,811	30 (19–45)	46	31
^***a***^Based on 2006 census data (Statistics Canada 2013). ^***b***^Defined by ICD-10-CA diagnostic code for appendicitis and a concurrent procedural code for appendectomy.								

We evaluated potential confounding by other air pollutants by using two-pollutant models adjusted for NO_2_ or PM_2.5_ during the same exposure periods as O_3_. Stratified analyses were used to estimate associations between ambient O_3_ exposure and appendicitis according to age (≤ 20, 20–39, and ≥ 40 years), sex, season [spring (March–May), summer (June–August), autumn (September–November), and winter (December–February)], and appendicitis phenotype (perforated versus nonperforated). Stratified models were compared using Cochran’s Q statistic.

We performed several sensitivity analyses. We excluded observations from Halifax to evaluate the impact of missing O_3_ data (missing for approximately 20% of days in Halifax compared with < 1% of days for the other 11 cities) on the overall pooled risk estimate. We also conducted a sensitivity analysis that included all cases with an ICD-10-CA diagnostic code for appendicitis (i.e., not restricted to those with also a procedural code). We also conducted analyses with exposure defined based on 24-hr mean O_3_ concentrations instead of daily 1-hr maximum concentrations, and analyses of associations with an exposure contrast of 10 ppb instead of 16 ppb (the IQR). In addition, we estimated associations adjusting for temperature and humidity during the same exposure periods as O_3_, instead of adjusting for temperature and humidity on the event or referent days only.

All statistical analyses were conducted in SAS (version 9.2; SAS Institute Inc., Cary, NC, USA). In all instances, a *p-*value < 0.05 was considered statistically significant. The study was approved by the Conjoint Health Research Ethics Board at the University of Calgary, who also granted a waiver of consent due to anonymized administrative data. Our study was conducted in accordance with the Strengthening of the Reporting of Observational Studies in Epidemiology (STROBE) statement (Vandenbroucke et al. 2007).

## Results

A total of 35,811 patients were classified as appendicitis cases based on the presence of both an ICD-10-CA diagnostic code for appendicitis and a procedural code for appendectomy in the 12 Canadian cities between 2004 and 2008. The median age at diagnosis was 30 years (IQR = 19–45 years), 54% were male, and 31% had perforated appendicitis (Table 1). The median daily maximum O_3_ concentration for the 12 cities was 33.3 ppb (IQR = 16 ppb).

**Figure 1:**
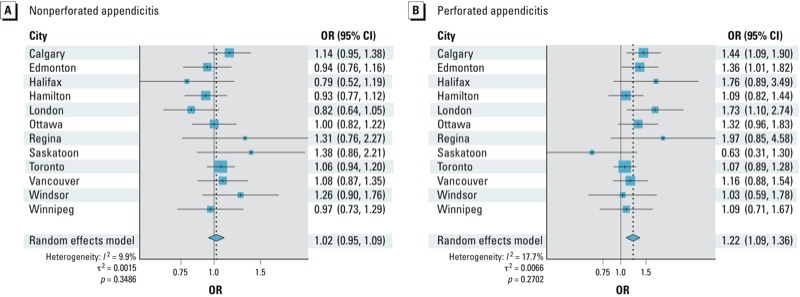
Forest plots for pooled and city-specific ORs (95% CI) for a 16-ppb increase in the 7-day average daily 1-hr maximum O_3_ concentration and nonperforated (*A*) and perforated appendicitis (*B*). The ORs of appendicitis in association with a 16-ppb increase in the daily maximum O_3_ concentration adjusted for mean temperature and relative humidity were estimated by conditional logistic regression.

The 7-day average daily maximum O_3_ concentration was positively associated with appendicitis in the pooled analysis (OR = 1.07; 95% CI: 1.02, 1.13) (Table 2), with little evidence of heterogeneity across the cities (*p* = 0.89) [see Supplemental Material, Table S1 (http://dx.doi.org/10.1289/ehp.1206085)]. Exposure was more strongly associated with perforated appendicitis, with ORs increasing as the period of exposure increased from 3 to 7 days (3-day OR = 1.11; 95% CI: 1.01, 1.23, 5-day OR = 1.15; 95% CI: 1.04, 1.27, 7-day OR = 1.22; 95% CI: 1.09, 1.36) (Table 2). In contrast, O_3_ exposure was not associated with nonperforated appendicitis (7-day OR = 1.02; 95% CI: 0.95, 1.09) (Table 2). The ORs for perforated cases were significantly different from corresponding ORs for nonperforated cases for all averaging periods (all *p* < 0.05). Forest plots for city-specific risk estimates of the 7-day average stratified by perforated and nonperforated appendicitis are presented in Figure 1. The OR for the association between the 7-day average of O_3_ and perforated appendicitis was > 1 for all cities except for Saskatoon (OR = 0.63; 95% CI: 0.31, 1.30). Heterogeneity was not statistically significant for the 7-day average for nonperforated (*p* = 0.48) and perforated appendicitis (*p* = 0.29) (see Supplemental Material, Table S1).

**Table 2:** Stratified analyses of association [OR (95% CI)] between daily maximum O_3_ exposures and appendicitis cases in 12 cities of Canada (2004–2008).

Model^*a*^	*n*	Same-day	1-day lag	Cumulative average daily maximum O_3_ concentration (IQR = 16 ppb)		
				3-day average	5-day average	7-day average
All appendicitis	35,811	1.00 (0.96, 1.04)	1.03 (1.00, 1.11)	1.03 (0.99, 1.08)	1.04 (0.99, 1.10)	1.07 (1.02, 1.13)
Appendicitis phenotype						
Nonperforated	24,730	1.00 (0.95, 1.06)	1.01 (0.97,1.05)	1.00 (0.94, 1.06)	0.99 (0.92, 1.07)	1.02 (0.95, 1.09)
Perforated	11,081	0.98 (0.93, 1.04)	1.07 (1.01, 1.14)	1.11 (1.01, 1.23)	1.15 (1.04, 1.27)	1.22 (1.09, 1.36)
Age (years)						
≤ 20	10,313	0.93 (0.88, 0.99)	0.97 (0.92, 1.02)	0.96 (0.88, 1.04)	1.00 (0.90, 1.11)	1.06 (0.96, 1.17)
21–39	13,474	1.00 (0.95, 1.05)	1.05 (1.00, 1.10)	1.04 (0.97, 1.11)	1.06 (0.98, 1.14)	1.08 (0.99, 1.18)
≥ 40	12,024	1.06 (0.97, 1.16)	1.05 (1.00, 1.11)	1.09 (1.01, 1.17)	1.06 (0.98, 1.15)	1.08 (0.99, 1.19)
Sex						
Male	19,509	0.99 (0.95, 1.04)	1.03 (0.99, 1.07)	1.03 (0.98, 1.09)	1.04 (0.98, 1.11)	1.05 (0.98, 1.13)
Female	16,302	1.00 (0.95, 1.06)	1.02 (0.98, 1.07)	1.03 (0.96, 1.10)	1.05 (0.95, 1.16)	1.11 (1.00, 1.22)
Season (all appendicitis)						
Spring	8,991	0.99 (0.91, 1.07)	1.01 (0.95, 1.07)	0.97 (0.89, 1.06)	0.96 (0.87, 1.06)	0.99 (0.89, 1.11)
Summer	9,504	0.96 (0.90, 1.02)	0.99 (0.94, 1.05)	1.02 (0.95, 1.10)	1.07 (0.98, 1.16)	1.08 (0.97, 1.21)
Autumn	9,038	0.99 (0.89, 1.10)	1.05 (0.98, 1.12)	1.04 (0.93, 1.16)	1.03 (0.92, 1.16)	1.08 (0.96, 1.22)
Winter	8,278	1.06 (0.98, 1.15)	1.08 (1.00, 1.17)	1.11 (1.00, 1.23)	1.07 (0.95, 1.20)	1.10 (0.95, 1.28)
Season (perforated appendicitis)						
Spring	2,668	1.04 (0.91, 1.18)	1.03 (0.87, 1.22)	1.10 (0.94, 1.29)	1.12 (0.93, 1.34)	1.19 (0.97, 1.46)
Summer	2,899	0.88 (0.79, 0.99)	0.99 (0.89, 1.09)	1.01 (0.88, 1.16)	1.12 (0.96, 1.30)	1.21 (1.02, 1.44)
Autumn	2,879	0.95 (0.81, 1.11)	1.14 (1.01, 1.28)	1.16 (0.95, 1.42)	1.19 (0.93, 1.51)	1.28 (0.96, 1.71)
Winter	2,635	1.05 (0.92, 1.21)	1.13 (0.97, 1.32)	1.13 (0.93, 1.38)	1.09 (0.82, 1.45)	1.09 (0.77, 1.55)
O_3_ + NO_2_ (all appendicitis)	34,335	1.01 (0.97, 1.05)	1.02 (0.99, 1.06)	1.04 (0.99, 1.09)	1.06 (1.01, 1.11)	1.08 (1.02, 1.14)
O_3_ + NO_2_ (perforated appendicitis)	10,736	1.01 (0.94, 1.08)	1.07 (1.00, 1.14)	1.13 (1.02, 1.25)	1.15 (1.04, 1.27)	1.17 (1.07, 1.28)
O_3_ + PM_2.5_ (all appendicitis)	34,335	1.01 (0.97, 1.04)	1.02 (0.99, 1.06)	1.03 (0.99, 1.08)	1.03 (0.98, 1.09)	1.06 (1.00, 1.13)
O_3_ + PM_2.5_ (perforated appendicitis)	10,736	1.00 (0.94, 1.06)	1.06 (1.00, 1.13)	1.09 (0.99, 1.20)	1.10 (1.00, 1.20)	1.12 (1.02, 1.23)
Perforated appendicitis defined only by diagnostic code^*b*^	13,014	1.06 (0.94, 1.07)	1.08 (1.02, 1.14)	1.12 (1.02, 1.24)	1.14 (1.04, 1.25)	1.20 (1.08, 1.33)
Halifax excluded
All appendicitis	34,335	1.01 (0.97, 1.04)	1.03 (1.00, 1.06)	1.04 (1.00, 1.08)	1.05 (1.00, 1.10)	1.08 (1.02, 1.14)
Perforated appendicitis	10,736	0.99 (0.93,1.05)	1.08 (1.01, 1.15)	1.12 (1.01, 1.25)	1.15 (1.03, 1.29)	1.21 (1.08, 1.35)
^***a***^Conditional logistic regression estimated the odds of appendicitis in association with a 16-ppb increase in the daily maximum O_3_ concentration adjusted for mean temperature and relative humidity on the same day as admission for appendicitis. ^***b***^Original definition includes an ICD-10-CA diagnostic code for appendicitis and a procedural code for appendectomy.						

The pooled OR for the 7-day average did not vary significantly (all *p* > 0.05) when stratified according to age group, sex, or season (Table 2). Pooled ORs for all appendicitis and perforated appendicitis were comparable with those for the population as a whole when Halifax was excluded (Table 2). Pooled ORs for both outcomes also were similar when adjusted for NO_2_ or PM_2.5_ in two-pollutant models. Our findings did not substantially change when adjusted for temperature and humidity during the same exposure period as O_3_ (instead of temperature and humidity on the index or referent days) (7-day average for perforated appendicitis: OR = 1.19; 95% CI: 1.06, 1.35) or when estimated for 24-hr mean O_3_ concentrations instead of daily 1-hr maximum concentrations (7-day average for perforated appendicitis OR = 1.18; 95% CI: 1.07, 1.30). Consistent with expectations, associations were weaker when estimated for a 10-ppb increase in daily 1-hr maximum O_3_ (7-day average for perforated appendicitis OR = 1.13; 95% CI: 1.05, 1.21) instead of an IQR (16 ppb) increase [Supplemental Material, Table S2 (http://dx.doi.org/10.1289/ehp.1206085)].

## Discussion

In this multicity study, short-term exposure to ambient O_3_ was associated with an increased number of hospital visits for appendicitis. The findings were robust across a number of sensitivity analyses and consistent with a prior single-city study (Kaplan et al. 2009). Associations with O_3_ were evident for perforated appendicitis, but not nonperforated appendicitis. We estimated an 11–22% increase in perforated appendicitis with every 16-ppb increase in daily 1-hr maximum O_3_ levels when averaged over the previous 3 to 7 days.

While the pooled relative risk estimates were modest in magnitude, our findings are consistent with previously reported associations between O_3_ and asthma (Villeneuve et al. 2007). That study of nearly 58,000 asthma visits to emergency departments in Edmonton demonstrated that an increase of 24 ppb of the 5-day average of O_3_ exposure was associated with an 8% increase in asthma exacerbations (Villeneuve et al. 2007). Our pooled estimates for associations with all appendicitis were consistent with a previous single-city study for Calgary, one of the cities included in the present analysis (Kaplan et al. 2009). Two previous studies found no association between air pollution and appendicitis (McGowan et al. 2002; Ponka and Virtanen 1996). However, McGowan et al. (2002) conducted a time-series analysis and only studied particulate matter, whereas Ponka and Virtanen (1996) analyzed their data using Poisson regression modeling and did not evaluate a multiday cumulative average of exposure of O_3_.

We did not observe statistically significant departures from homogeneity across the 12 cities studied. However, O_3_ concentrations were inversely associated with perforated appendicitis in Saskatoon, in contrast with the other 11 cities. Differences among the cities could reflect differences in the temporal or spatial variability of O_3_ levels for individual cities. NAPS monitoring stations are generally located in areas with air pollution levels that are expected to be representative of background concentrations in a city. Although averaging measurements from multiple fixed monitoring sites into one daily value for the entire city may misclassify exposures at the individual level (Sajani et al. 2010), this potential bias is likely to be low for O_3_ because O_3_ levels are spatially homogeneous across a region (Chen et al. 2007). Missing data may have contributed to variability among cities; however, associations were essentially unchanged when the city of Halifax, where daily O_3_ levels were missing for approximately 20% of the study period, was excluded from the analysis.

Our findings were robust across numerous different approaches to analyzing the data. Although appendicitis is more often diagnosed in young persons (Addiss et al. 1990; Luckmann and Davis 1991) and in the male sex (Addiss et al. 1990; Luckmann and Davis 1991), neither age nor sex appeared to influence associations between O_3_ and appendicitis in our study population. O_3_ levels are lower in winter months (Chen et al. 2007), when people are also less likely to be exposed to ambient O_3_ because of increased time spent indoors, thus potentially increasing the likelihood of exposure misclassification. Associations were inconsistent when stratified by season. For example, during the summer, perforated appendicitis was negatively associated with exposure on the same day (OR = 0.88; 95% CI: 0.79, 0.99), but positively associated with exposure averaged over the 7 previous days (OR = 1.21; 95% CI: 1.02, 1.44). However, season-stratified associations should be interpreted cautiously because the sample sizes were reduced and the differences between seasons were not statistically significant. Finally, although air pollutants are often correlated (Chen et al. 2007), estimates from two-pollutant models adjusted for NO_2_ or PM_2.5_ were comparable to adjusted estimates, suggesting that associations between O_3_ and perforated appendicitis were not confounded by these other air pollutants.

O_3_ may selectively influence the pathogenesis of perforated as compared with nonperforated appendicitis. Although perforated appendicitis may result from a delay in diagnosing appendicitis (Bickell et al. 2006), emerging evidence suggests that perforated appendicitis also may represent a distinct disease phenotype (Andersson 1999; Ruber et al. 2010). For example, perforated appendicitis may have a divergent immunological pathogenesis [e.g., T helper (Th)-17 predominant] as compared with nonperforated appendicitis (Ruber et al. 2006, 2010). O_3_ exposure in humans induced a proinflammatory systemic response through stimulation of tumor necrosis factor, interleukin (IL)-6, and IL-8 (Bosson et al. 2007; Paulesu et al. 1991; Srebot et al. 2009; Thompson et al. 2010). In addition, in an animal study, exposure to air pollutants elevated IL-8 and IL-17 levels in the small and large bowel and altered the intestinal microflora of mice (Kish et al. 2013). Further, increased intestinal permeability in mice exposed to particulate matter appeared to result from increased inflammation, disruption of tight junctions, and death of epithelial cells (Mutlu et al. 2011). Potential effects of air pollution on proinflammatory immune responses, and on the host microbiome, could contribute to the development of perforated appendicitis.

Alternatively, the differential association of O_3_ with perforated versus nonperforated appendicitis may be noncausal. Case definitions of appendicitis were based on ICD-10-CA coding of an administrative database. A validation study comparing ICD coding of appendicitis against pathology-proven appendicitis suggested high sensitivity (> 90%), but approximately 15% of cases were false positives (e.g., misclassifying incidental appendectomy of a normal appendix as appendicitis) (Kareemi et al. 2012). Nondifferential misclassification error of the disease outcome may bias the risk estimates. Cases coded as perforated appendicitis are less likely to be false-positive or false-negative than cases coded as nonperforated appendicitis (Kareemi et al. 2012). Thus, the association between O_3_ and perforated appendicitis may represent the relationship between O_3_ and appendicitis when outcome misclassification is minimized. Additional studies of pathology-proven nonperforated and perforated appendicitis cases are needed to confirm that associations with O_3_ are specific to perforated appendicitis, rather than overall appendicitis.

Several other limitations should be considered:
O_3_ exposure was regionally assigned rather being measured at the patient level.
The measurement of O_3_ levels was restricted to ambient levels that may not represent indoor exposures.
Multiple comparisons were performed in stratified analyses and, thus, some significant findings may have occurred by chance.
Small sample sizes in some of the cities (e.g., Saskatoon) may have led to spurious associations.

Although persons serve as their own controls in a case-crossover study design, we cannot rule out residual confounding by time varying factors. Socioeconomic status may influence the development of perforated as compared to nonperforated appendicitis. Recent studies have reported that socioeconomic status was not associated with the risk of perforated appendicitis in large cohorts in the United States and Canada. (Lee et al. 2011; Livingston and Fairlie 2012; To and Langer 2010). In addition in Canada, access to health care is less influenced by socioeconomic status because of universal health coverage in Canada. Nonetheless, additional studies are needed to determine whether the association between O_3_ and perforated appendicitis is modified by socioeconomic status.

O_3_ may not be a causal factor but may instead represent a proxy marker of one or more causal exposures. However, associations between O_3_ and perforated appendicitis were not appreciably altered by adjustment for NO_2_ or PM_2.5_ in two-pollutant models.

## Conclusions

We conducted a multicity study that used a validated case definition and controlled for potential confounders through the case-crossover study design, adjustment for meteorological effects, and the selection of referent intervals using a time-stratified approach. Our findings suggest that short-term ambient O_3_ exposure increases the risk of perforated appendicitis. Consequently, air pollution may be a contributing factor to the pathogenesis of appendicitis.

## Supplemental Material

(246 KB) PDF
