# Supplementary material for: Ambient Ozone Concentrations and the Risk of Perforated and Nonperforated Appendicitis: A Multicity Case-Crossover Study
Source: Environ Health Perspect. 2013 Jul 11;121(8):939–43. doi: 10.1289/ehp.1206085 (PMC3734492; doi:10.1289/ehp.1206085)
Supplement: (246 KB) PDF [file ehp.1206085.s001.pdf]

Supplemental Material

Ambient Ozone Concentrations and the Risk of Perforated and Nonperforated Appendicitis: A Multicity Case-Crossover Study

Gilaad G Kaplan, Divine Tanyingoh, Elijah Dixon, Markey Johnson, Amanda J. Wheeler, Robert P. Myers, Stefania Bertazzon, Vineet Saini, Karen Madsen, Subrata Ghosh, and Paul J Villeneuve

Table of Contents

Page 2 – Supplemental Material, Table S1.

Page 3 - Supplemental Material, Table S2.

Supplemental Material, Table S1. P-values for heterogeneity between 12 cities for the main models.

**Models**	**Same-Day P-Value Heterogeneity**	**1-Day Lag P-Value Heterogeneity**	**3-Day Average P-Value Heterogeneity**	**5-Day Average P-Value Heterogeneity**	**7-Day Average P-Value Heterogeneity**
All Appendicitis	0.15	0.49	0.46	0.31	0.89
Appendicitis Phenotype					
Nonperforated	0.16	0.37	0.15	0.08	0.35
Perforated	0.58	0.33	0.14	0.23	0.27

Supplemental Material, Table S2. Odds ratios (95% CI) based on alternative models of the association between ozone exposures and appendicitis adjusted for temperature and humidity in 12 cities of Canada (2004 – 2008).

			Cumulative Exposure		
**Model**	**Same-Day**	**1-Day Lag**	**3-Day Average**	**5-Day Average**	**7-Day Average**
Maximum ozone, temperature and humidity on day of admission[a]					
All Appendicitis	1.00 (0.96, 1.04)	1.03 (1.00, 1.11)	1.03 (0.99, 1.08)	1.04 (0.99, 1.10)	1.07 (1.02, 1.13)
Appendicitis Phenotype					
Nonperforated	1.00 (0.95, 1.06)	1.01 (0.97,1.05)	1.00 (0.94, 1.06)	0.99 (0.92, 1.07)	1.02 (0.95, 1.09)
Perforated	0.98 (0.93, 1.04)	1.07 (1.01, 1.14)	1.11 (1.01, 1.23)	1.15 (1.04, 1.27)	1.22 (1.09, 1.36)
Maximum ozone, temperature and humidity during the same period as ozone[a]					
All Appendicitis	1.00 (0.96, 1.04)	1.02 (0.99, 1.05)	1.03 (0.99, 1.07)	1.03 (0.97, 1.09)	1.06 (1.00, 1.12)
Appendicitis Phenotype					
Nonperforated	1.00 (0.96, 1.06)	1.00 (0.96, 1.04)	1.00 (0.94 ,1.07)	0.99 (0.91, 1.07)	1.01 (0.94, 1.08)
Perforated	0.98 (0.93, 1.04)	1.07 (1.00, 1.14)	1.10 (0.99, 1.21)	1.13 (1.02, 1.27)	1.19 (1.06, 1.35)
24-hour mean ozone, temperature and humidity on day of admission[a]					
All Appendicitis	1.00 (0.97, 1.04)	1.02 (1.00, 1.05)	1.03 (0.99, 1.07)	1.03 (0.99, 1.08)	1.06 (1.02, 1.11)
Appendicitis Phenotype					
Nonperforated	1.00 (0.96, 1.05)	1.01 (0.97, 1.04)	1.00 (0.95, 1.05)	0.99 (0.93, 1.06)	1.02 (0.96, 1.08)
Perforated	0.98 (0.94, 1.03)	1.06 (1.01, 1.12)	1.10 (1.01, 1.19)	1.12 (1.03, 1.23)	1.18 (1.07, 1.30)
10 ppb increase in maximum ozone, temperature and humidity on day of admission[b]					
All Appendicitis	1.00 (0.98, 1.03)	1.02 (1.00, 1.03)	1.02 (0.99, 1.05)	1.02 (0.99, 1.06)	1.05 (1.01, 1.08)
Appendicitis Phenotype					
Nonperforated	1.00 (0.97, 1.03)	1.00 (0.98, 1.03)	1.00 (0.96, 1.04)	1.00 (0.95, 1.04)	1.01 (0.97, 1.06)
Perforated	0.99 (0.95, 1.02)	1.04 (1.01, 1.08)	1.07 (1.01, 1.14)	1.09 (1.02, 1.16)	1.13 (1.05, 1.21)

[a]Conditional logistic regression model of the odds of appendicitis in association with a 16 ppb increase in the daily maximum ozone concentration or 24-hour mean ozone adjusted for mean temperature and relative humidity as indicated.

[b]Conditional logistic regression model of the odds of appendicitis in association with a 10 ppb increase in the daily maximum ozone concentration adjusted for mean temperature and relative humidity on day of admission.
